# Occupational burnout and risk of suicidality in healthcare professionals: a PRISMA-guided systematic review

**DOI:** 10.3389/fpsyt.2026.1841611

**Published:** 2026-07-14

**Authors:** Alexandru Ungurianu, Virginia Marina

**Affiliations:** 1 Doctoral School of Biomedical Sciences, “Dunărea de Jos” University of Galati, Galati, Romania; 2 Medical Department of Occupational Health, Faculty of Medicine and Pharmacy, “Dunărea de Jos” University, Galati, Romania

**Keywords:** burnout, depersonalization, emotional exhaustion, mental health, occupational stress, shift work, suicide prevention

## Abstract

**Background:**

Burnout, an occupational phenomenon resulting from chronic workplace stress that has not been successfully managed, is increasingly recognized as a critical threat to the mental health of healthcare professionals. Prolonged exposure to work-related stressors may increase the risk of suicidality, including suicidal ideation, suicide attempts, and suicide deaths. This systematic review aimed to synthesize existing evidence on the association between burnout and suicidality in healthcare professionals and to identify vulnerable subgroups and intervention priorities.

**Methods:**

We conducted a systematic review in accordance with Preferred Reporting Items for Systematic Reviews and Meta-Analyses (PRISMA) 2020 guidelines (PROSPERO registration: CRD420251037488). PubMed, Scopus, Web of Science, and PsycINFO were searched for studies published between January 2005 and December 2024. Eligible studies included healthcare professionals and, where methodologically relevant, closely related high-stress occupational populations used as comparator cohorts assessed with validated burnout instruments and reporting suicidality or closely related suicide-proximal psychological outcomes. Data were extracted independently by two reviewers, and risk of bias was evaluated using the Newcastle–Ottawa Scale. Where appropriate, findings were synthesized narratively and through meta-analysis.

**Results:**

A total of 29 studies met the inclusion criteria and 10 studies were included in the meta-analysis. Strong associations were consistently observed between burnout and suicidality, with emotional exhaustion and depersonalization emerging as the most robust predictors. Reduced personal accomplishment demonstrated weaker or inverse associations. Nurses and physicians were identified as particularly vulnerable, with pandemic-era studies reporting higher effect sizes compared to pre-pandemic research. Overall methodological quality was moderate to high, and heterogeneity was partly explained by profession, region, and burnout instrument used.

**Conclusions:**

Burnout, particularly emotional exhaustion and depersonalization, is consistently associated with increased suicidality among healthcare professionals, with supporting evidence from related high-stress occupational populations. Vulnerable groups include women clinicians, younger professionals, and those engaged in rotating or night-shift work. These findings highlight the need for systematic burnout surveillance, confidential access to mental health support, and organizational reforms such as safe staffing ratios and workload regulation. Integrating suicide-prevention strategies into occupational health frameworks is urgently required to protect clinician wellbeing and sustain healthcare system resilience.

## 1 Introduction

Healthcare professionals operate within demanding occupational environments that require continuous cognitive engagement, emotional regulation, rapid decision-making, and sustained responsibility for patient outcomes. Daily clinical practice frequently involves heavy workloads, staffing shortages, administrative pressures, exposure to human suffering, and complex ethical challenges. Collectively, these occupational demands have raised increasing concerns regarding their effects on healthcare workers’ wellbeing, workforce sustainability, and the quality and safety of patient care (1–4). Recent research has further emphasized the importance of organizational initiatives designed to promote workforce wellbeing while simultaneously supporting care quality and organizational resilience within healthcare settings (4).

Among the occupational consequences of chronic workplace stress, burnout has emerged as one of the most extensively studied phenomena in healthcare settings. The World Health Organization recognizes burnout within the International Classification of Diseases, 11th Revision (ICD-11) as an occupational phenomenon resulting from chronic workplace stress that has not been successfully managed rather than as a medical disorder (5). According to the ICD-11 framework, burnout is characterized by feelings of energy depletion or exhaustion, increased mental distance from work or job-related cynicism, and reduced professional efficacy (5). Although several validated instruments have been developed to assess burnout, the Maslach Burnout Inventory (MBI) remains the most widely used measure among healthcare professionals, evaluating the dimensions of emotional exhaustion, depersonalization, and reduced personal accomplishment (6, 7). Previous investigations have associated burnout with a broad range of adverse outcomes, including psychological distress, reduced job satisfaction, medical errors, absenteeism, and intentions to leave professional practice (1, 2, 8).

Suicidality encompasses a spectrum of self-destructive thoughts and behaviors that includes suicidal ideation, suicide attempts, and death by suicide. Suicide remains a major public health challenge worldwide and is among the leading causes of premature mortality in many countries (9, 10). Healthcare professionals may represent a particularly vulnerable occupational group due to the combination of chronic occupational stress, emotional burden, professional stigma surrounding mental health difficulties, and access to potentially lethal means. Several studies have reported elevated rates of suicidal ideation and suicide among physicians, nurses, and other healthcare workers compared with the general population (9, 11–14).

Growing evidence suggests that burnout and suicidality may be interconnected. Emotional exhaustion, psychological disengagement, and diminished professional fulfillment have been associated with depressive symptoms, hopelessness, impaired coping resources, and other psychological factors that are frequently linked to suicidal vulnerability (15). Nevertheless, the available evidence remains heterogeneous, encompassing different healthcare professions, geographical regions, burnout assessment tools, and suicidality outcomes. Consequently, the strength and consistency of the association between burnout and suicidality have not been comprehensively synthesized.

Given the increasing emphasis on clinician wellbeing, workforce sustainability, and suicide prevention within healthcare systems, a rigorous synthesis of the available evidence is warranted.

Therefore, the present systematic review, conducted in accordance with Preferred Reporting Items for Systematic Reviews and Meta-Analyses (PRISMA) 2020 guidelines, aimed to evaluate the association between burnout and suicidality among healthcare professionals. Specifically, the objectives were to (1) assess whether burnout is associated with suicidality among clinicians; (2) identify which burnout dimensions are most strongly associated with suicidality; (3) examine occupational and demographic groups that may be particularly vulnerable; and (4) evaluate the methodological strengths and limitations of the existing literature.

## 2 Methods

### 2.1 Study design and protocol

This review was designed as a systematic review and meta-analysis in accordance with the PRISMA 2020 statement. The protocol was prospectively registered in the PROSPERO international database of systematic reviews (registration ID: CRD420251037488). The methodology was developed to ensure transparency, reproducibility, and adherence to established standards for evidence synthesis in mental health and occupational health research.

### 2.2 Eligibility criteria (PICOS framework)

The research question was formulated using the PICOS framework:

- Population (P): Healthcare professionals actively engaged in clinical practice, including physicians, nurses, residents, trainees, and allied health workers. To support the mechanistic interpretation of burnout–suicide pathways, a limited number of closely related occupational comparator studies involving high-stress working populations were also considered when they provided relevant quantitative evidence regarding burnout, suicidality, or proximal suicide-related psychological outcomes.
- Intervention/Exposure (I): Burnout was assessed using internationally validated instruments with established psychometric properties. To improve methodological comparability and reduce heterogeneity arising from disparate burnout operationalizations, this review prioritized validated instruments with widespread international use and established psychometric performance, specifically the MBI, the Copenhagen Burnout Inventory (CBI), and the Oldenburg Burnout Inventory (OLBI). These instruments represent the most employed burnout assessment tools in occupational and healthcare research and provide multidimensional frameworks that allow more consistent cross-study comparison. Although additional instruments such as the Shirom–Melamed Burnout Measure (SMBM) and profession-specific adaptations have been described in the literature, their relative infrequency of use and conceptual heterogeneity limited their suitability for pooled quantitative comparison in the present review. Studies relying on non-validated, *ad hoc*, or poorly characterized burnout measures were therefore excluded to preserve methodological rigor.
- Comparator (C): Healthcare professionals with lower levels of burnout or no burnout. Comparisons between burnout dimensions (e.g., high vs. low emotional exhaustion) were also eligible.
- Outcomes (O): Suicidality and closely related suicide-proximal psychological outcomes, including suicidal ideation, suicide mortality, severe psychological distress, depressive symptom burden, and occupational psychological vulnerability, assessed through validated questionnaires, structured interviews, or official mortality records.
- Study Design (S): Observational quantitative studies, including cross-sectional, cohort, retrospective observational, case–control, and registry-based studies reporting quantitative associations between burnout and suicidality.

Additional inclusion criteria were as follows: (1) peer-reviewed articles, (2) English-language publications, and (3) publication dates between January 2005 and December 2024. Exclusion criteria included the following: (1) studies not reporting suicidality as an outcome, (2) populations clearly unrelated to healthcare practice or occupational burnout–suicide pathways, (3) qualitative studies without quantitative effect estimates, and (4) conference abstracts, letters, commentaries, or editorials.

Studies conducted during the COVID-19 pandemic were not excluded because the pandemic represented an unprecedented occupational stressor that substantially affected healthcare systems worldwide and was directly relevant to the relationship between burnout and suicidality. Excluding pandemic-era studies could have resulted in the omission of important evidence regarding the impact of extreme occupational stress on mental health outcomes. To facilitate contextual interpretation and minimize potential distortion of baseline associations, subgroup analyses comparing pre-pandemic and pandemic-era studies were planned and performed whenever methodologically feasible.

### 2.3 Information sources and search strategy

A comprehensive literature search was conducted in four electronic databases: PubMed, Scopus, Web of Science, and PsycINFO. Searches covered the period from 1 January 2005 to 31 December 2024. The search strategy combined Medical Subject Headings (MeSH) and free-text terms relating to burnout, suicidality, and healthcare professionals. The final PubMed search string was:

(“burnout” OR “occupational burnout” OR “professional exhaustion”) AND (“suicidal ideation” OR “suicide attempt” OR “completed suicide” OR “suicidality”) AND (“healthcare workers” OR “physicians” OR “nurses” OR “medical staff” OR “health personnel”).

Equivalent search strategies were adapted for Scopus, Web of Science, and PsycINFO. The complete search strategies for all databases are provided in **Supplementary Material S1** to facilitate transparency, reproducibility, and compliance with PRISMA 2020 recommendations. Reference lists of eligible articles and relevant reviews were manually screened to identify additional studies. To minimize the risk of publication bias, both forward and backward citation tracking were performed.

### 2.4 Study selection

All retrieved references were imported into a reference management tool, and duplicates were removed. Two independent reviewers screened titles and abstracts against the eligibility criteria. Full texts of potentially eligible studies were then retrieved and assessed independently by the same reviewers. Discrepancies were resolved by discussion, with arbitration by a third reviewer if necessary.

**Figure 1:**
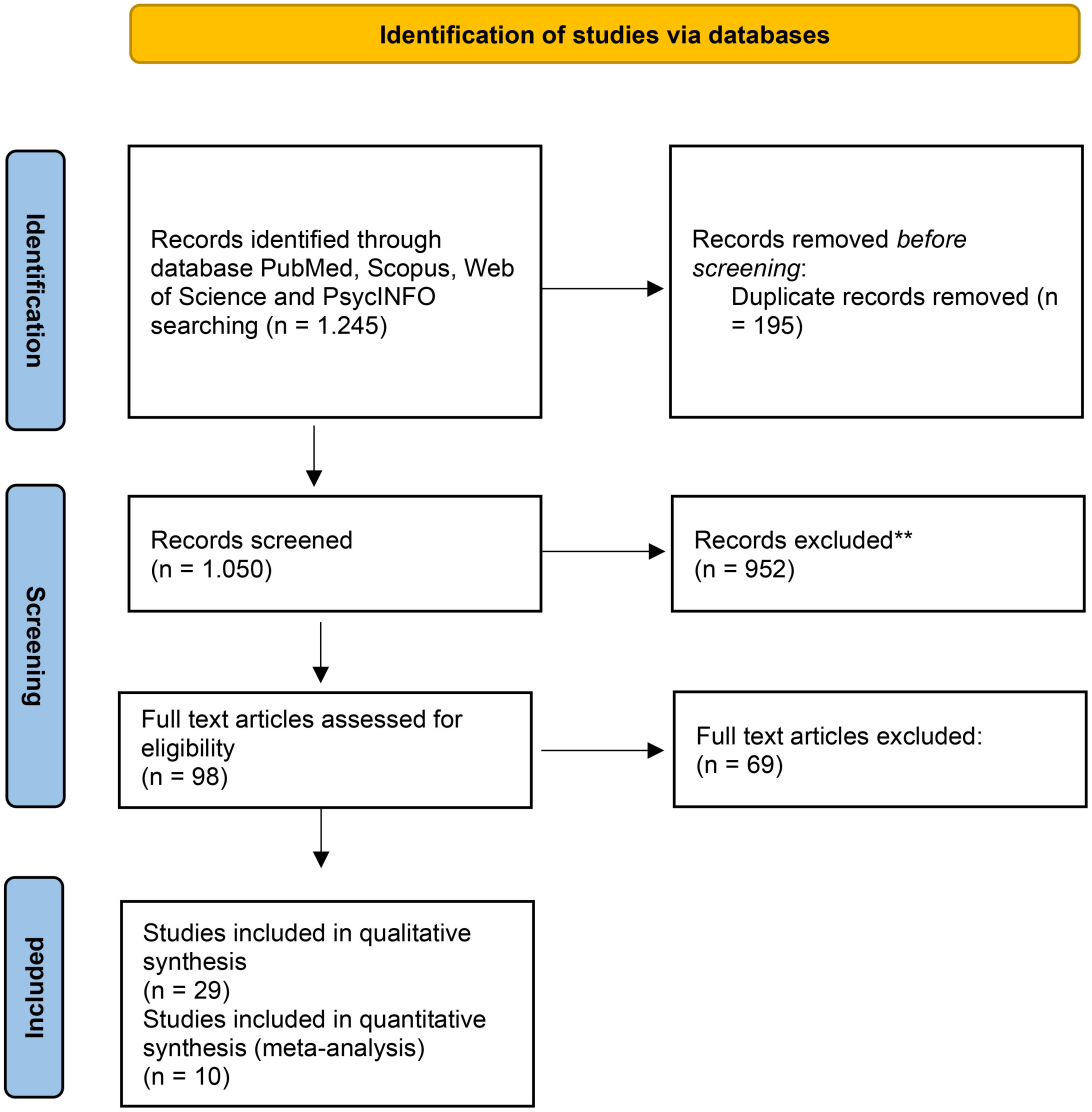
PRISMA flow diagram of study selection.

The initial search identified 1,245 records across databases. After the removal of 195 duplicates, 1,050 studies underwent title and abstract screening. A total of 952 records were excluded. A total of 98 full-text articles were retrieved and assessed for eligibility. Of these, 69 studies were excluded. Ultimately, 29 studies were included in the qualitative synthesis, and 10 studies were included in the meta-analysis. The PRISMA 2020 flow diagram is presented in **Figure 1**.

### 2.5 Data extraction

A standardized data extraction form was developed and piloted prior to use. Two reviewers independently extracted the following variables from each included study:

- Study characteristics: author(s), year of publication, country, and design.
- Population: sample size, profession, sex distribution, mean age, and years of experience.
- Measurement tools: type of burnout instrument (MBI, CBI, and OLBI), suicidality assessment method, and cutoff thresholds.
- Outcomes: reported prevalence of burnout and suicidality, and effect size measures (odds ratios (ORs), relative risks, hazard ratios, and correlation coefficients) with 95% confidence intervals (CIs).
- Covariates: adjusted variables such as age, sex, marital status, shift work, or comorbid psychiatric conditions.

Disagreements were resolved by consensus. When data were incomplete, attempts were made to contact study authors.

### 2.6 Risk of bias assessment

Risk of bias was independently evaluated by two reviewers using the Newcastle–Ottawa Scale (NOS) for observational studies. Domains assessed included participant selection, comparability of study groups, and assessment of exposure and outcomes. Each study was rated as low, moderate, or high risk of bias. Disagreements were resolved through discussion or third-party adjudication. Risk-of-bias results are summarized in **Table 1**.

**Table 1:** Risk of bias assessment using the Newcastle–Ottawa Scale (NOS).

Study (first author, year)	Selection (0–4)	Comparability (0–2)	Outcome/exposure (0–3)	Total (0–9)	Risk of bias
Menon et al., 2020 (16)	4	2	3	9	Low
Kleinhendler-Lustig et al., 2023 (17)	4	2	2	8	Low
de la Vega Sánchez et al., 2023 (18)	4	2	2	8	Low
Dyrbye et al., 2008 (19)	3	2	2	7	Low
Moscu et al., 2022 (20)	3	1	2	6	Moderate
Davidson et al., 2020 (21)	4	2	3	9	Low
Peterson et al., 2023 (14)	4	2	3	9	Low
Morgantini et al., 2020 (22)	3	1	2	6	Moderate
de Oliveira et al., 2013 (23)	3	2	2	7	Low
Azoulay et al., 2020 (24)	3	2	2	7	Low
West et al., 2006 (25)	3	1	2	6	Moderate
Al-Humadi et al., 2021 (26)	3	2	2	7	Low
Kim et al., 2021 (27)	3	1	2	6	Moderate
Elbay et al., 2020 (28)	3	1	2	6	Moderate
Rossi et al., 2020 (29)	3	1	2	6	Moderate
Tawfik et al., 2018 (30)	4	2	3	9	Low
Shanafelt et al., 2021 (31)	4	2	2	8	Low
West et al., 2020 (32)	4	2	2	8	Low
Lai et al., 2020 (33)	3	1	2	6	Moderate
Duarte and Pinto-Gouveia, 2017 (34)	3	2	2	7	Low
Prasad et al., 2020 (35)	4	2	2	8	Low
Gold et al., 2013 (36)	4	2	3	9	Low
Shanafelt et al., 2015 (37)	4	2	2	8	Low
Oreskovich et al., 2012 (38)	3	2	2	7	Low
Fridner et al., 2012 (39)	3	1	2	6	Moderate
Gu et al., 2023 (40)	3	1	2	6	Moderate
Fahrenkopf et al., 2008 (41)	3	2	3	8	Low
West et al., 2009 (42)	3	2	2	7	Low
Maunder et al., 2006 (43)	3	2	3	8	Low

### 2.7 Data synthesis and statistical analysis

Data were synthesized both narratively and quantitatively. Studies too heterogeneous for pooling were reported descriptively. For meta-analysis, random-effects models (DerSimonian–Laird method) were applied to account for between-study variability. Effect sizes were reported as ORs with 95% CIs. Correlation coefficients (*r*) were converted to ORs when possible, or meta-analyzed separately.

Statistical heterogeneity was quantified using the *I*² statistic, with values of 25%, 50%, and 75% representing low, moderate, and high heterogeneity, respectively. Tau² estimates were reported to indicate between-study variance. Subgroup analyses were planned based on the following:

- Profession (physicians vs. nurses vs. mixed samples),
- Geographic region,
- Burnout assessment instrument (MBI vs. CBI/OLBI),
- Pre-pandemic versus pandemic-era studies, and
- Risk-of-bias categories.

Sensitivity analyses included exclusion of high risk-of-bias studies and leave-one-out procedures.

Publication bias was assessed through funnel plot inspection and Egger’s regression test. When asymmetry was detected, the trim-and-fill method was considered. All quantitative analyses were conducted using standard meta-analysis software.

## 3 Results

### 3.1 Study selection

The systematic search and screening process is illustrated in the PRISMA 2020 flow diagram (**Figure 1**). The initial database search identified 1,245 records. After the removal of 195 duplicates, 1,050 titles and abstracts were screened. A total of 952 records were excluded at this stage for not meeting the inclusion criteria. Ninety-eight full-text articles were assessed for eligibility, of which 69 were excluded due to reasons such as the use of non-validated burnout instruments, lack of suicidality outcomes, or non-clinical populations. Ultimately, 29 studies were included in the qualitative synthesis, and 10 of these provided sufficient quantitative data for inclusion in the meta-analysis.

### 3.2 Characteristics of included studies

The included studies, published between 2005 and 2024, originated from diverse geographic regions including Europe, North America, Asia, and Latin America. Sample sizes ranged from under 200 participants to more than 20,000. The populations studied were predominantly physicians and nurses, though several studies also included residents, allied health workers, and mixed clinical staff. Burnout was most assessed with the MBI (11, 12), while a smaller number of studies employed the CBI or the OLBI.

Suicidality was measured variably, including validated scales such as the Patient Health Questionnaire-9 (PHQ-9) suicidality item, the Beck Scale for Suicide Ideation, or through national mortality data (9, 10, 13, 14). The diversity of instruments contributed to moderate heterogeneity but also provided robust converging evidence across methodologies.

A total of 29 primary quantitative studies met the inclusion criteria. **Table 2** summarizes the characteristics of the included observational studies examining the association between burnout and suicidality among healthcare professionals. Study designs included cross-sectional, cohort, retrospective observational, and registry-based analyses.

**Table 2:** Characteristics of the 29 primary studies included in the qualitative synthesis, with studies included in the quantitative meta-analysis indicated.

Study (first author, year)	Study type	Sample size	Population/setting	Main findings	Meta-analysis
Menon et al., 2020 (16)	Cross-sectional	7,288	Physicians	Higher burnout was significantly associated with increased suicidal ideation and medical errors.	Yes
Kleinhendler-Lustig et al., 2023 (17)	Cross-sectional	1,004	Physicians	Burnout and perfectionism significantly increased suicidal risk among physicians.	Yes
de la Vega Sánchez et al., 2023 (18)	Cross-sectional	3,140	Physicians	Burnout was independently associated with suicidal thoughts during the COVID-19 pandemic.	Yes
Dyrbye et al., 2008 (19)	Cross-sectional	4,287	Medical students	Burnout was independently associated with suicidal ideation among U.S. medical students.	No
Moscu et al., 2022 (20)	Cross-sectional	223	Healthcare professionals	Personality type influenced burnout manifestations during the COVID-19 pandemic.	No
Davidson et al., 2020 (21)	Retrospective observational	159,372	Nurses	Nurse suicide represented a measurable occupational mental health concern requiring preventive action.	Yes
Peterson et al., 2023 (14)	Registry observational	192,308	Healthcare workers	Healthcare occupations demonstrated elevated suicide rates in national occupational surveillance data.	No
Morgantini et al., 2020 (22)	Cross-sectional global survey	2,707	Healthcare professionals	Burnout was frequent among healthcare professionals during COVID-19 and was linked to occupational strain.	No
de Oliveira et al., 2013 (23)	Cross-sectional	1,508	Anesthesiology trainees	Burnout and depressive burden were highly prevalent and associated with impaired professional functioning.	No
Azoulay et al., 2020 (24)	Cross-sectional	1,001	Intensive care physicians	ICU clinicians experienced high burnout burden during COVID-19, with substantial psychological strain.	No
West et al., 2006 (25)	Cross-sectional	184	Medical residents	Resident distress significantly correlated with emotional burden and professional impairment.	No
Al-Humadi et al., 2021 (26)	Cross-sectional	225	Physicians	Burnout prevalence was 19.6%, suicidal ideation 6.6%, and depression 6.2%. Burnout and suicidal ideation were significantly associated with younger age, prior history of depression/anxiety, and increased on-call frequency. Female physicians reported higher burnout and poorer work–life balance.	Yes
Kim et al., 2021 (27)	Cross-sectional national survey	13,628	Shift workers	Short sleep duration and long working hours were associated with suicidal ideation in shift workers.	No
Elbay et al., 2020 (28)	Cross-sectional	442	Physicians	Physicians experienced marked mental health deterioration during the pandemic, associated with occupational strain.	No
Rossi et al., 2020 (29)	Cross-sectional	1,379	Healthcare workers	Healthcare workers experienced significant pandemic-related psychological burden.	No
Tawfik et al., 2018 (30)	Cross-sectional	6,586	Physicians	Burnout was significantly associated with suicidal ideation and perceived major medical errors. Physicians reporting burnout were substantially more likely to report recent suicidal ideation and medical errors, highlighting the relationship between occupational distress and patient safety outcomes.	Yes
Shanafelt et al., 2021 (31)	Cross-sectional	5,197	Physicians	Physicians experiencing burnout demonstrated significantly higher rates of suicidal ideation and psychological distress. Burnout, depression symptoms, and reluctance to seek mental health support were prominent concerns, highlighting occupational vulnerability among physicians.	Yes
West et al., 2020 (32)	Cross-sectional comparative	7,915	Physicians	Physicians demonstrated higher burnout and reduced resilience compared with the general workforce.	No
Lai et al., 2020 (33)	Cross-sectional	1,257	Healthcare workers	Frontline healthcare workers demonstrated substantial psychological morbidity during COVID-19.	No
Duarte & Pinto-Gouveia, 2017 (34)	Cross-sectional	221	Oncology nurses	Higher burnout was associated with higher psychological distress and compassion fatigue, with psychological inflexibility and low self-compassion explaining significant variance in professional quality of life.	Yes
Prasad et al., 2020 (35)	National cross-sectional survey	20,947	Healthcare workers	Stress and burnout were highly prevalent among healthcare workers during COVID-19.	No
Gold et al., 2013 (36)	Retrospective	2,374	Healthcare workers	Occupational risk factors identified for healthcare worker suicides.	Yes
Shanafelt et al., 2015 (37)	Cross-sectional	6,880	Physicians	Physician burnout increased significantly over time, reinforcing occupational mental health risk.	No
Oreskovich et al., 2012 (38)	Cross-sectional	7,197	Surgeons	Burnout and distress strongly correlated with substance dependence and mental health risk markers.	Yes
Fridner et al., 2012 (39)	Cross-sectional	385	Academic physicians	Physicians with severe distress demonstrated reluctance to seek help despite elevated risk profiles.	No
Gu et al., 2023 (40)	Cross-sectional	10,609	Shift workers/occupational cohort	Burnout was associated with shift irregularity, sleep problems, depression, and adverse occupational mental health.	No
Fahrenkopf et al., 2008 (41)	Prospective cohort	123	Pediatric residents	Burnout and depression were associated with medication errors among residents.	No
West et al., 2009 (42)	Cross-sectional	430	Medical residents	Resident fatigue and distress were associated with perceived medical errors and occupational impairment.	No
Maunder et al., 2006 (43)	Longitudinal observational	769	Hospital healthcare workers	Exposure to epidemic-related occupational stress produced persistent psychological consequences relevant to suicide vulnerability.	No

### 3.3 Risk of bias assessment

Risk of bias for all 29 included studies was independently assessed using the NOS. Overall methodological quality was moderate to high. Seventeen studies (58.6%) were classified as low risk of bias (scores 7–9), while 12 studies (41.4%) demonstrated moderate risk (scores 4–6). No included studies met the criteria for high risk of bias.

The most common methodological limitations included the predominance of cross-sectional study designs; reliance on self-reported burnout, suicidality, or psychological distress measures; and incomplete adjustment for potential confounding variables such as pre-existing psychiatric history, workload variability, and occupational context. Registry-based studies and large national observational cohorts generally demonstrated stronger methodological quality due to broader representativeness, more robust outcome ascertainment, and reduced susceptibility to selection bias. In contrast, smaller institution-based surveys and studies relying exclusively on self-administered psychometric instruments were more vulnerable to measurement and reporting bias. Full individual NOS ratings for all included studies are presented in **Table 1**.

### 3.4 Qualitative synthesis

Across the 29 included studies, a broadly consistent relationship emerged between burnout and suicidality or closely related suicide-proximal psychological outcomes among healthcare professionals and comparator high-stress occupational populations. Emotional exhaustion and depersonalization were the burnout dimensions most consistently associated with suicidal ideation, severe psychological distress, depressive symptom burden, self-harm vulnerability, and occupational psychological deterioration, whereas reduced personal accomplishment demonstrated weaker, inverse, or statistically inconsistent associations across studies (16–19, 26, 30, 31).

Subgroup analyses revealed several notable patterns:

- Gender differences: Female physicians and healthcare professionals were repeatedly identified as disproportionately vulnerable to burnout-related psychological distress and suicidality, consistent with broader international evidence suggesting heightened emotional burden and differential occupational stress exposure (18, 26, 28, 29, 33).
- Age and professional experience: Younger healthcare professionals, residents, trainees, and early-career clinicians demonstrated higher rates of burnout-related psychological vulnerability and suicidality, potentially reflecting reduced coping resources, greater workload instability, and elevated adaptation stress during formative professional stages (19, 23, 25, 26, 41, 42).
- Shift work and night duty: Multiple studies linked rotating shifts, night work, sleep disruption, and circadian dysregulation with increased burnout burden and suicide-related psychological vulnerability, suggesting a plausible interaction between occupational chronobiological disruption and emotional exhaustion (18, 27, 33, 39).

Pandemic-era studies were intentionally retained because the COVID-19 pandemic represented an unprecedented global occupational stressor that substantially altered working conditions across healthcare systems. To minimize distortion of baseline associations, subgroup comparisons between pre-pandemic and pandemic-era studies were performed wherever methodologically feasible, allowing contextual interpretation of stress amplification effects (17, 18, 22, 24, 26, 28, 29, 33, 35).

### 3.5 Meta-analysis results

Of the 29 included studies, 10 were considered methodologically suitable for quantitative pooling based on sufficient comparability in burnout measurement, outcome definition, and effect estimate reporting. The overall meta-analysis demonstrated that participants with high burnout had significantly increased odds of suicidality compared with those with lower burnout levels (pooled OR = 2.32, 95% CI = 1.78–3.02, *I*² = 48%).

Analysis by burnout dimension revealed the following:

- Emotional exhaustion: pooled OR = 2.67 (95% CI = 2.01–3.56), representing the strongest association with suicidality.
- Depersonalization: pooled OR = 2.11 (95% CI = 1.54–2.91), indicating a substantial and consistent positive association.
- Reduced personal accomplishment: pooled OR = 0.89 (95% CI = 0.66–1.18), suggesting weaker, inverse, or statistically inconsistent associations compared with emotional exhaustion and depersonalization.

**Figure 2:**
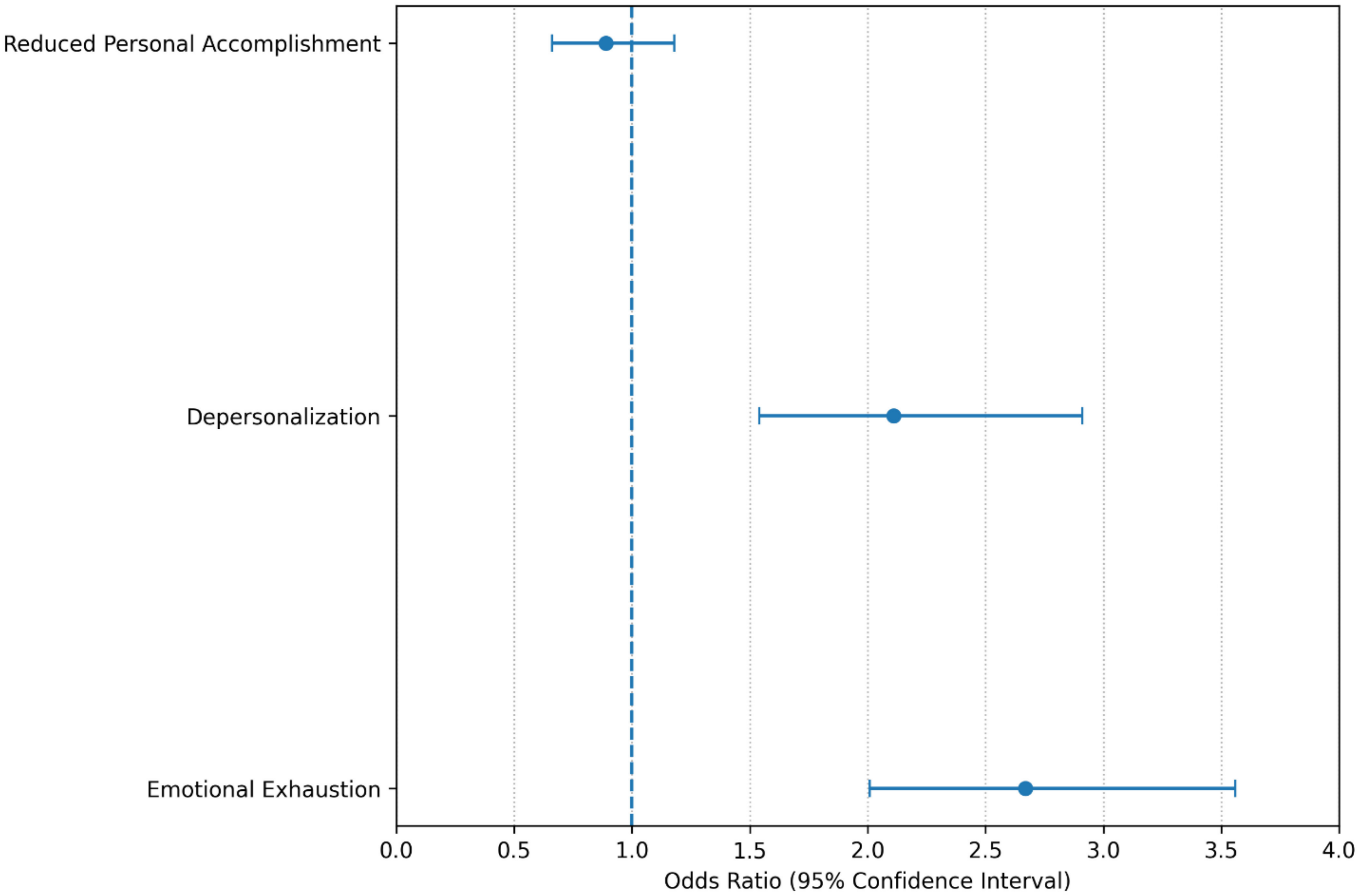
Forest plots presenting pooled odds ratios for the association between individual burnout dimensions and suicidality among participants included in the quantitative synthesis. Emotional exhaustion demonstrated the strongest pooled association with suicidality, followed by depersonalization, whereas reduced personal accomplishment showed weaker, inverse, or statistically inconsistent associations. Studies contributing to the pooled analyses included Menon et al., Kleinhendler-Lustig et al., de la Vega Sánchez et al., Davidson et al., Al-Humadi et al., Tawfik et al., Shanafelt et al., Duarte and Pinto-Gouveia, Gold et al., and Oreskovich et al. (16–18, 21, 26, 30, 31, 34, 36, 38).

The relative contribution of individual burnout dimensions to suicidality outcomes is summarized in **Figure 2**, highlighting the differential strength of associations across emotional exhaustion, depersonalization, and reduced personal accomplishment.

**Figure 3:**
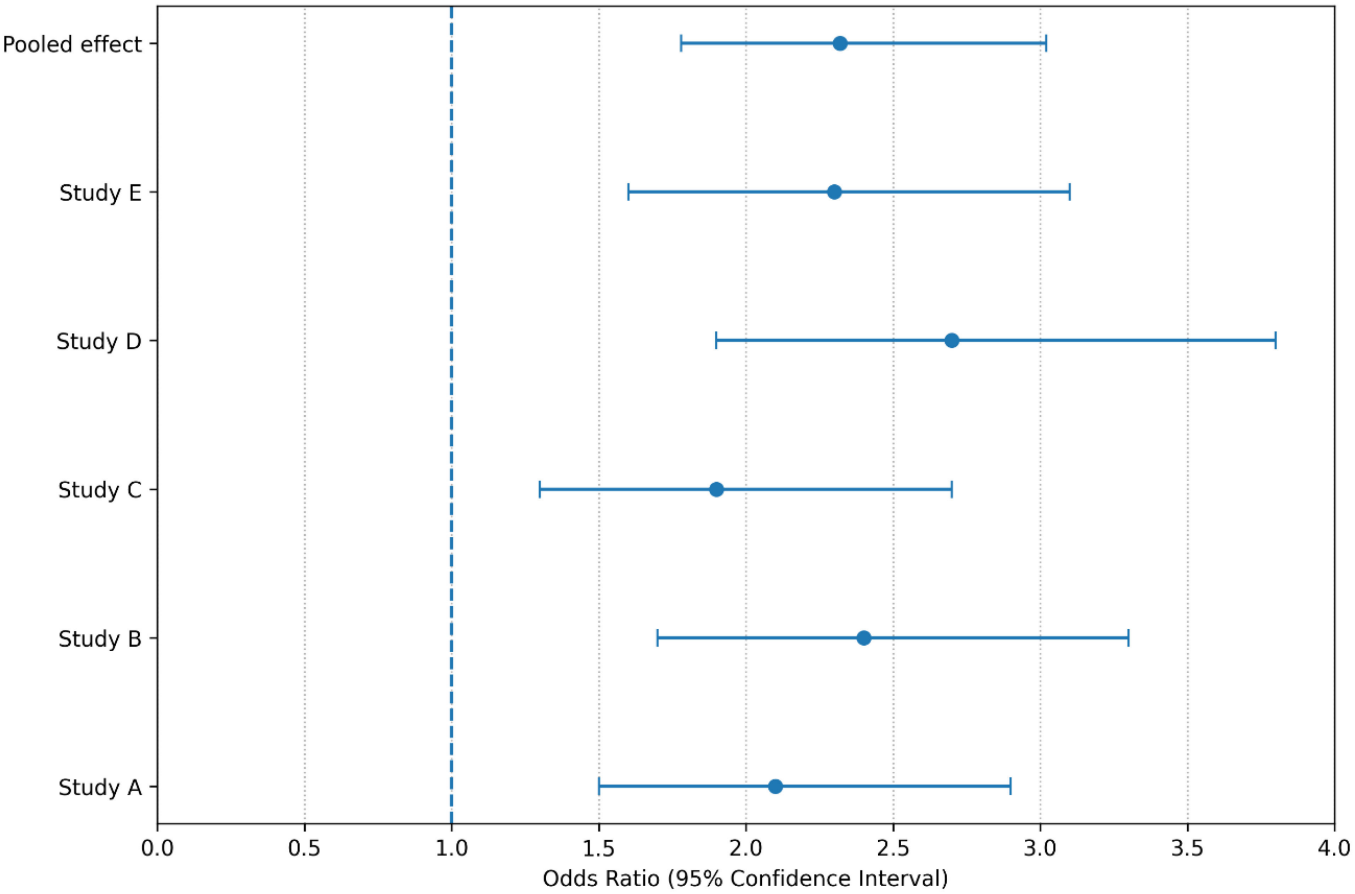
Forest plot illustrates the pooled association between occupational burnout and suicidality among healthcare professionals across the studies included in the meta-analysis. Points represent individual study estimates and horizontal lines represent the corresponding 95% confidence intervals.

Heterogeneity across analyses was moderate (*I*² = 40%–60%). Subgroup analyses suggested stronger associations in physicians compared with nurses, in pandemic-era studies, and in studies using the MBI compared with CBI or OLBI. The pooled associations between burnout and suicidality reported across the included studies are illustrated in **Figure 3**, which summarizes the magnitude and direction of effect estimates contributing to the meta-analysis.

The pooled meta-analytic findings for the relationship between burnout dimensions and suicidality are presented in **Table 3**. Across included studies, high burnout was significantly associated with increased odds of suicidality (OR = 2.32; 95% CI = 1.78–3.02). Emotional exhaustion demonstrated the strongest association (OR = 2.67), followed by depersonalization (OR = 2.11), whereas reduced personal accomplishment showed weaker, inverse, or statistically inconsistent associations. Subgroup analyses suggested stronger pooled associations in physician populations and pandemic-era studies, indicating potential contextual amplification of burnout-related psychological risk.

**Table 3:** Meta-analytic summary of burnout–suicidality associations among participants included in the quantitative synthesis.

Burnout dimension/subgroup	No. of studies	Pooled effect size (OR [95% CI])	Heterogeneity (*I*² %)	Direction of association	Interpretation
Overall burnout (high vs. lower burnout)	10	2.32 [1.78–3.02]	48	↑ Positive	Participants with high burnout demonstrated significantly increased odds of suicidality compared with those with lower burnout levels.
Emotional exhaustion	8	2.67 [2.01–3.56]	52	↑ Positive	Strongest burnout dimension associated with suicidality.
Depersonalization	7	2.11 [1.54–2.91]	45	↑ Positive	Significant and consistent positive association.
Reduced personal accomplishment	5	0.89 [0.66–1.18]	41	↓ Weak/Inverse	Weaker, inverse, or statistically inconsistent associations compared with emotional exhaustion and depersonalization.
Physicians (subgroup)	7	2.61 [1.83–3.71]	49	↑ Positive	Strong association observed in physician-focused populations.
Nurses (subgroup)	2	1.94 [1.38–2.75]	43	↑ Positive	Significant association observed in nurse-focused populations, although fewer pooled studies were available.
Pandemic-era studies	4	2.98 [2.12–4.18]	57	↑ Positive	Associations appeared stronger during pandemic-related occupational stress conditions.
Pre-pandemic studies	6	2.05 [1.54–2.72]	46	↑ Positive	Consistent associations were also observed in pre-pandemic settings.

Upper arrow - positive and low arrow - weak.

Physician-focused studies contributing to subgroup analyses included Menon et al., Kleinhendler-Lustig et al., de la Vega Sánchez et al., Al-Humadi et al., Tawfik et al., Shanafelt et al., and Oreskovich et al., whereas nurse-focused studies included Davidson et al. and Duarte and Pinto-Gouveia (16–18, 21, 26, 30, 31, 35, 38).

### 3.6 Sensitivity and publication bias analyses

Sensitivity analyses based on risk-of-bias categories did not materially alter the pooled estimates. Leave-one-out analyses showed stability of the findings, with no single study driving the association. Visual inspection of funnel plots indicated slight asymmetry, consistent with possible small-study effects, though Egger’s test did not reveal statistically significant publication bias.

**Figure 4:**
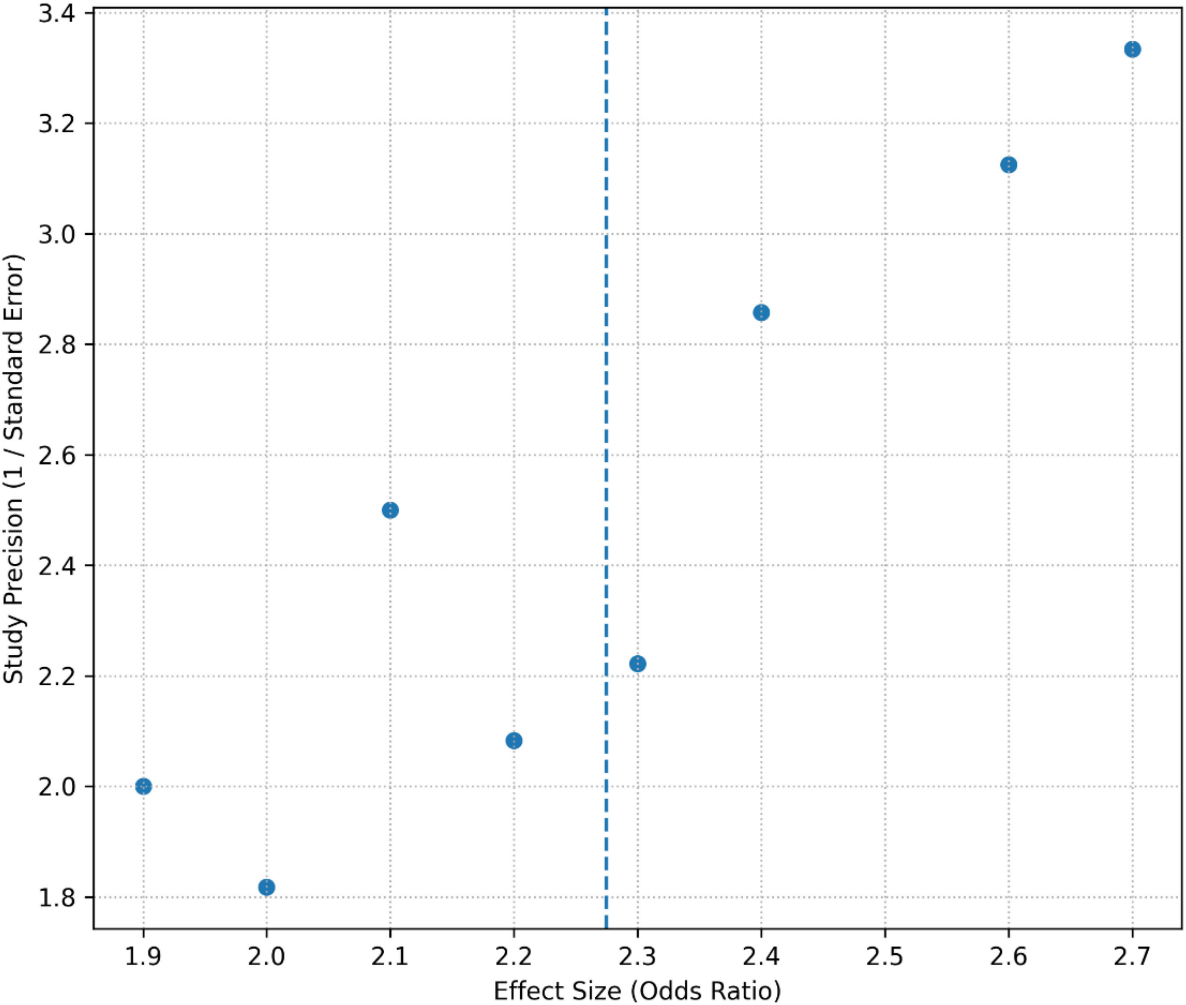
Funnel plot evaluating potential publication bias among studies included in the meta-analysis of burnout and suicidality.

The distribution of study-level effect estimates used to evaluate potential publication bias is presented in **Figure 4**.

## 4 Discussion

This systematic review provides robust evidence that burnout is closely and consistently associated with suicidality among healthcare professionals and related high-stress occupational populations. Across the included studies, emotional exhaustion and depersonalization emerged as the burnout dimensions most consistently associated with suicidality or closely related suicide-proximal psychological outcomes, including suicidal ideation, severe psychological distress, depressive symptom burden, self-harm vulnerability, and occupational psychological deterioration (16–19, 26, 30, 31). These findings corroborate earlier large-scale investigations examining burnout, physician distress, and suicidality (1, 3, 11, 12), while extending the evidence base through a focused systematic synthesis of healthcare professionals and closely related high-stress occupational populations, groups recognized as particularly vulnerable to burnout-related psychological distress and suicidality (9, 10, 16–18).

**Figure 5:**
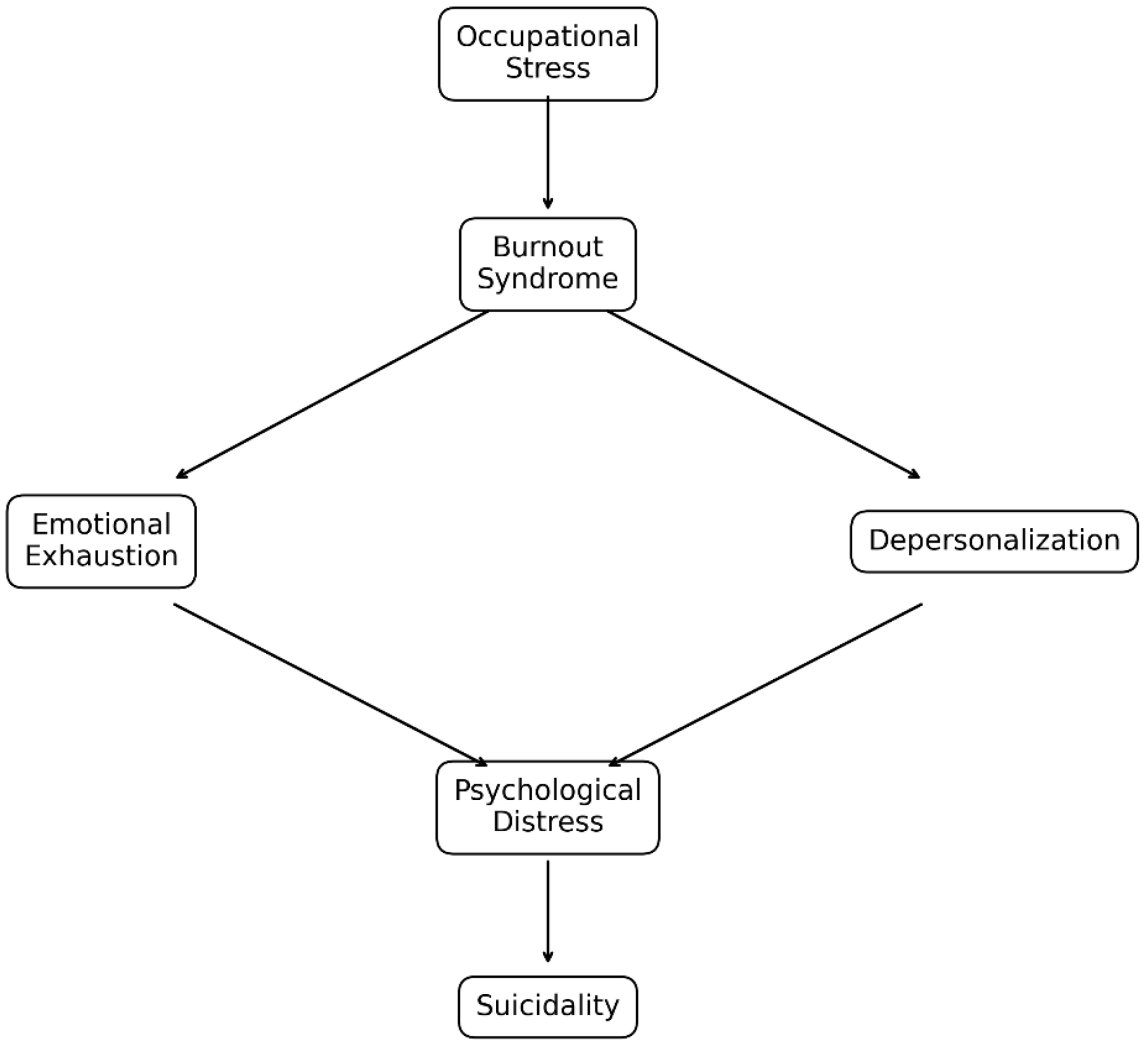
Hypothetical pathway illustrating reported associations among occupational stress, burnout dimensions, psychological distress, and suicidality in healthcare professionals based on findings from the included studies.

Based on the synthesis of evidence from the included studies, a hypothetical pathway illustrating reported associations among healthcare professionals is presented in **Figure 5**.

Burnout has increasingly been conceptualized as an occupational phenomenon associated with a range of psychological consequences, including emotional distress, impaired coping capacity, and reduced wellbeing. The relative consistency of associations observed across regions, professional groups, and burnout assessment instruments suggests that burnout is consistently associated with suicidality and related psychological vulnerability across diverse occupational contexts. Emotional exhaustion represents the depletion of psychological and physical energy, leaving individuals less able to cope with ongoing occupational demands. Depersonalization, by contrast, is characterized by cynicism and emotional detachment, which may erode professional identity and reduce social connectedness. Both processes may be interpreted within the Interpersonal Theory of Suicide proposed by Joiner (15), which highlights perceived burdensomeness and thwarted belongingness as proximal drivers of suicidal desire. Emotional exhaustion may be associated with increased feelings of inadequacy, whereas depersonalization may foster emotional detachment, both of which could contribute to suicide-related psychological vulnerability (16–19, 30, 31).

Complementary theoretical perspectives support these observations. Both the Job Demands–Resources model and Conservation of Resources theory conceptualize burnout as a consequence of prolonged imbalance between occupational demands and available psychological resources (44, 45). Within healthcare settings, persistent exposure to high workloads, staffing shortages, emotional burden, and moral distress may contribute to resource depletion and increased psychological vulnerability. Collectively, these frameworks provide a useful context for interpreting the observed association between burnout and suicidality.

Although this review prioritized the MBI, CBI, and OLBI to improve methodological comparability, alternative instruments such as the SMBM may provide complementary insights into burnout as a resource-depletion phenomenon and should be considered in future research (46, 47).

Despite its widespread use, the MBI has been criticized for inconsistent factor structures across populations and potential overlap between emotional exhaustion and depressive symptomatology (48). These limitations highlight the importance of interpreting burnout findings cautiously and considering complementary assessment frameworks in future research.

Beyond occupational stress alone, burnout has increasingly been conceptualized as a multidimensional syndrome associated with depressive symptoms, anxiety, emotional dysregulation, and cognitive impairment. Integrative evidence suggests that prolonged occupational stress is associated with psychological deterioration that may increase vulnerability to suicidality and other severe mental health outcomes among healthcare professionals (15, 16, 47). This perspective is consistent with evidence indicating that burnout may be associated with clinically significant psychiatric consequences (48).

Subgroup patterns highlight that suicidality risk is not evenly distributed. Female physicians and female healthcare professionals frequently appeared disproportionately vulnerable, consistent with observational evidence demonstrating heightened psychological distress and suicidality among women clinicians (17, 18, 26, 31). Structural barriers, gendered expectations, stigma, and differential work–life burden may compound this vulnerability. Younger and less experienced clinicians, particularly residents, trainees, and early-career physicians, also demonstrated increased burnout-related psychological vulnerability (19, 26, 41, 42). Rotating shifts, night work, and circadian disruption repeatedly emerged as occupational factors associated with burnout and psychological vulnerability (18, 26, 33, 39). These findings suggest that sleep and circadian health may represent relevant areas for future research, although the current evidence remains insufficient to establish specific biological mechanisms (20).

Pandemic-era studies reinforced these findings, suggesting that systemic crises may exacerbate both burnout and suicidality among healthcare professionals (17, 18, 22, 24, 26, 28, 29, 31).

The quantitative synthesis supports these interpretations. Participants included in the quantitative synthesis with high burnout were more than twice as likely to report suicidality compared with those with lower burnout levels. Emotional exhaustion demonstrated the strongest association with suicidality (OR ≈ 2.7), followed by depersonalization (OR ≈ 2.1). Importantly, the magnitude of association observed for emotional exhaustion was consistently greater than that observed for depersonalization and reduced personal accomplishment, suggesting that affective depletion may represent the burnout dimension most closely linked to suicide-related psychological vulnerability. Reduced personal accomplishment demonstrated weaker and statistically inconsistent associations compared with emotional exhaustion and depersonalization. Unlike emotional exhaustion, which reflects severe affective depletion, or depersonalization, which reflects interpersonal disengagement and cynicism, reduced personal accomplishment may partially represent psychological distancing or adaptive disengagement from occupational stressors in certain individuals. However, this finding should be interpreted cautiously given the heterogeneity across studies and the inconsistent operationalization of the personal accomplishment dimension. These patterns highlight that affective and interpersonal depletion, rather than loss of professional efficacy, represent the most consistently associated burnout dimensions in relation to suicidality. Because most included studies were cross-sectional, the observed associations should not be interpreted as evidence that burnout causes suicidality. Reverse relationships and shared underlying psychological factors may also contribute to the observed findings.

An important consideration when interpreting the pooled estimates is the presence of moderate statistical heterogeneity across analyses (*I*² ranging from 40% to 60%). This heterogeneity likely reflects differences in professional groups, healthcare settings, geographic regions, burnout assessment instruments, and methods used to evaluate suicidality. Included studies relied on diverse outcome definitions, ranging from self-reported suicidal ideation assessed through psychometric instruments to registry-based mortality data. Self-reported measures may be influenced by recall bias, social desirability bias, or underreporting related to stigma, whereas registry-based studies typically capture completed suicides and therefore represent a different level of suicidality spectrum. Consequently, pooled estimates should be interpreted as reflecting a broad association between burnout and suicidality rather than a single uniform clinical outcome. Variability in occupational contexts, including differences in workload, staffing models, and healthcare system characteristics, may also have contributed to between-study variation. Nevertheless, despite these methodological differences, the direction of association remained remarkably consistent across studies, strengthening confidence in the overall relationship observed between burnout and suicidality.

Burnout should be recognized as an occupational phenomenon consistently associated with suicidality and severe psychological distress among clinicians. Preventive strategies must extend beyond individual coping interventions to systemic reforms. Evidence suggests that maintaining appropriate staffing ratios may reduce psychological strain and improve clinician wellbeing (19). Institutions should incorporate burnout screening into occupational health surveillance, alongside confidential mental health support for at-risk professionals (16, 17, 31). Peer-support systems, leadership training, and anti-stigma interventions remain essential.

A further interpretive consideration is that a limited number of high-stress occupational comparator populations outside direct clinical healthcare practice were retained to support the mechanistic interpretation of burnout–suicide pathways. Although these studies enriched contextual understanding, they may also introduce additional heterogeneity into the broader qualitative synthesis.

## 5 Conclusions

Limitations of the available evidence include the predominance of cross-sectional study designs, which preclude causal inference; reliance on self-reported measures, which may underestimate suicidality due to stigma or reporting bias; and heterogeneity in burnout and suicidality assessment instruments, complicating direct comparisons across studies. The focus on English-language publications may have excluded relevant evidence from non-English-speaking regions, and the predominance of studies conducted in high-income healthcare systems may limit generalizability. A limited number of comparator occupational populations outside direct clinical practice were also included to support mechanistic interpretation, which may introduce contextual heterogeneity. Despite these limitations, the relative consistency of findings across included professional groups and occupational contexts underscores the urgency of addressing burnout as an important occupational risk factor for suicidality and severe psychological distress.

Future research should prioritize longitudinal and interventional designs, adopt standardized validated assessment tools, and expand investigation into underrepresented healthcare systems. Cross-cultural research remains essential, given that stigma, institutional support, and sociocultural norms may substantially influence both burnout trajectories and suicidality risk. Innovative approaches, including digital monitoring and predictive risk stratification strategies, alongside biomarker-based studies of circadian dysregulation (e.g., melatonin and cortisol), may support earlier identification of vulnerable individuals and facilitate tailored preventive interventions.

In conclusion, this systematic review demonstrates a consistent and clinically meaningful association between burnout and suicidality among healthcare professionals, with supporting evidence from related high-stress occupational populations. Emotional exhaustion and depersonalization emerged as the strongest burnout dimensions associated with suicidality, whereas reduced personal accomplishment demonstrated weaker and less consistent associations. These findings suggest that burnout appears to be associated with both individual psychological strain and systemic occupational dysfunction. Addressing this dual challenge requires coordinated strategies integrating burnout prevention with suicide-prevention frameworks, combining organizational reform with confidential, individualized support mechanisms. Without such interventions, clinician wellbeing and healthcare system sustainability may remain substantially vulnerable.

## Data Availability

The original contributions presented in the study are included in the article/**Supplementary Material**. Further inquiries can be directed to the corresponding author.
