# Supplementary material for: Occupational burnout and risk of suicidality in healthcare professionals: a PRISMA-guided systematic review
Source: Front Psychiatry. 2026 Jul 14;17:1841611. doi: 10.3389/fpsyt.2026.1841611 (PMC13407641; doi:10.3389/fpsyt.2026.1841611)
Supplement: Supplementary file 1 [file Supplementaryfile1.docx]

**Supplementary Material S1: Complete Search Strategies Used in the Systematic Review**

**Database: PubMed**

("burnout" OR "occupational burnout" OR "professional exhaustion")
AND
("suicidal ideation" OR "suicide attempt" OR "completed suicide" OR "suicidality")
AND
("healthcare workers" OR "physicians" OR "nurses" OR "medical staff" OR "health personnel")

Search period:
January 1, 2005 – December 31, 2024

Language restriction:
English

**Database: Scopus**

TITLE-ABS-KEY
(
("burnout" OR "occupational burnout" OR "professional exhaustion")
AND
("suicidal ideation" OR "suicide attempt" OR "completed suicide" OR "suicidality")
AND
("healthcare workers" OR "physicians" OR "nurses" OR "medical staff" OR "health personnel")
)

**Database: Web of Science**

TS=
(
("burnout" OR "occupational burnout" OR "professional exhaustion")
AND
("suicidal ideation" OR "suicide attempt" OR "completed suicide" OR "suicidality")
AND
("healthcare workers" OR "physicians" OR "nurses" OR "medical staff" OR "health personnel")
)

**Database: PsycINFO**

(
("burnout" OR "occupational burnout" OR "professional exhaustion")
AND
("suicidal ideation" OR "suicide attempt" OR "completed suicide" OR "suicidality")
AND
("healthcare workers" OR "physicians" OR "nurses" OR "medical staff" OR "health personnel")
)

Search period: January 1, 2005 - December 31, 2024

Language restriction: English.
